# Wild rodents and insectivores as carriers of pathogenic *Leptospira* and *Toxoplasma gondii* in The Netherlands

**DOI:** 10.1002/vms3.255

**Published:** 2020-03-05

**Authors:** Inge M. Krijger, Ahmed A. A. Ahmed, Maria G. A. Goris, Jan B. W. J. Cornelissen, Peter W. G. Groot Koerkamp, Bastiaan G. Meerburg

**Affiliations:** ^1^ Wageningen University & Research , Livestock Research Wageningen The Netherlands; ^2^ Farm Technology Group Wageningen University & Research Wageningen The Netherlands; ^3^ Department of Medical Microbiology OIE and National Collaborating Centre for Reference and Research on Leptospirosis (NRL) Academic Medical Centre University of Amsterdam Amsterdam The Netherlands; ^4^ Wageningen BioVeterinary Research Lelystad The Netherlands; ^5^ Dutch Pest and Wildlife Expertise Centre (KAD) Wageningen The Netherlands

**Keywords:** leptospirosis, mice, pathogen–host relationship, rats, reservoir, zoonoses

## Abstract

Small mammals such as rodents can to carry zoonotic pathogens. Currently, there is impaired knowledge on zoonotic pathogens in rodents and insectivores in the Netherlands. This limits opportunities for preventive measures and complicates risk‐assessments for zoonotic transmission to humans. *Leptospira* spp. and *Toxoplasma gondii* are present on a list of prioritized emerging pathogens in the Netherlands and were therefore the focus of this study. Both pathogens have the ability to survive under moist environmental conditions. In total, a group of 379 small mammals (rodents & insectivores) were tested on pathogenic *Leptospira* spp., and 312 on *T. gondii*. Rodents and insectivores were trapped at various sites, but mostly on pig and dairy farms throughout the country. Over five percent of the animals (5.3%, *n* = 379) tested positive for *Leptospira* DNA, and five of the animals (1.6%, *n* = 312) tested were positive for *T. gondii* DNA. The animals positive for *T.gondii* were all brown rats and the ones for *Leptospira* spp. were various species. Our results show that insectivores and rodents might be used as an indicator for the environmental contamination and/or the contamination in wildlife for *Leptospira* spp.

## Graphical abstract

Over five percent of the rodents and insectivores (5.3%, *n* = 379) tested positive for *Leptospira* DNA and five of the animals (*n* = 312) tested were positive for *Toxoplasma gondii* DNA. *Leptospira* spp. and *T. gondii* are present in the Dutch population of wild small mammals, indicating the importance of the studies for these infectious agents. Both rodent ánd insectivore species might be considered as potential sources for human leptospirosis in The Netherlands and as an indicator for the environmental contamination and the contamination in, and of wildlife.

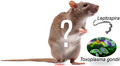

## 1 INTRODUCTION

Rodents and insectivores can be potential hosts for numerous zoonotic pathogens (Meerburg, Singleton, & Kijlstra, 2009). Thus, it is essential to monitor the pathogen presence in these small mammal populations. There is impaired knowledge (e.g. prevalence, geographic distribution, rodent species that are host) on rodent borne diseases in the Netherlands, which limits opportunities for preventive measures and complicates the assessment of risk of zoonotic transmission to humans. In order to increase the understanding on rodent‐borne pathogens in the Netherlands, we set up a study to assess the pathogen presence in common rodent and insectivore species from the Netherlands. Two important pathogens were selected from a list of prioritized emerging pathogens relevant for the Netherlands; (I) *Leptospira* spp., and (II) *Toxoplasma gondii*. Both pathogens are able to infect a wide range of species (Acha & Szyfres, 2003; Bharti et al., 2003; Levett, 2001; Newell et al., 2010; Opsteegh, 2011). The spirochaetal bacteria *Leptospira* spp. causes leptospirosis, which is an acute febrile disease in humans occurring worldwide (Bharti et al., 2003). The global burden of human leptospirosis is estimated on more than 60,000 deaths and over 1 million of severe leptospirosis cases in studies led by the World Health Organization (Costa et al., 2015; Torgerson et al., 2015; WHO, 2011). The bacterium is generally transmitted via direct or indirect contact with spirochetes secreted in the environment via the urine of infected reservoir animals (Hartskeerl, Collares‐Pereira, & Ellis, 2011). Hosts can be divided into reservoir and accidental hosts. Reservoir hosts are animal species which do not show symptoms after infection, and act as infection reservoir by lifelong shedding of leptospires in their urine and via parent–offspring transmission (Foley & Straub, 2017; Mwachui, Crump, Hartskeerl, Zinsstag, & Hattendorf, 2015). Accidental hosts shed only for a relative short period leptospires in their urine after infection, and these hosts develop severe or even lethal disease after infection (a.o. humans) (Fraga, Carvalho, Isaac, & Barbosa, 2015; Mwachui et al., 2015) Shedded leptospires have the ability to survive for prolonged periods of time in moist environments (Levett, 2001). Moreover, contaminated water is a serious risk for infection (Haake et al., 2002). One of the most important wildlife reservoir hosts are rodents (Faine, 1994; Terpstra, 1989). In the Netherlands brown rats (*Rattus norvegicus*) and voles (*Microtus arvalis*) are the most important reservoir species and infection sources for human leptospirosis (Fernandes et al., 2016; Guernier et al., 2016; Himsworth, Parsons, Jardine, & Patrick, 2013; Obiegala et al., 2017; Zilber et al., 2016; Zuerner, 2015). A publication from Hartskeerl and Terpstra (1996) from the Netherlands showed that other rodents species and some insectivores can also be reservoir hosts (hedgehogs (*Erinaceus europeanus*), muskrats (*Ondatra zibethicus*), house shrews (*Crocidura russula*) and house mice (*Mus musculus*)). Nevertheless, there is hardly scientific information available on the presence of *Leptospira* spp. in rodents and insectivores in the Netherlands. In 1934 a report was published on Leptospirosis in the Netherlands, mentioning a prevalence of *Leptospira* spp. in brown rats of 11%–56% (*n* = 60), emphasizing the differences between test‐locations (Schüffner, 1934). Research from 1992 on muskrats in the Netherlands found 7% (*n* = 327) positive on *Leptospira interrogans* (Steinen, Schuurman, Gravekamp, Korver, & Terpstra, 1992). More recently, a study on *Leptospira* spp. in brown rats found a prevalence of 42% (*n* = 150), with prevalences varying between geographical areas within the Netherlands (range of 33%–57%) (Maas et al., 2018). The National Institute for Public Health and the Environment in the Netherlands (RIVM) tested 189 mice on *Leptospira* spp. from between 2007 and 2015*,* and found 45.5% of *Apodemus sylvaticus* (*N* = 55), 73.3% of the tested *Microtus arvalis* (*n* = 60) and 41.8% of the tested *Myodus glareolus* (*n* = 74) mice positive for *Leptospira* spp. (Uiterwijk et al., 2016).

Besides potential carriage of *Leptospira* spp., rodents and insectivores can also be infected with *T. gondii*, a protozoan parasite (Dubey, 2014, 2016; Kim & Weiss, 2008; Krijger, Cornelissen, Wisselink, & Meerburg, 2014; Robert‐Gangneux & Dardé, 2012). Rodents have been suggested to be reservoirs of infection for cats, pigs and dogs (Dubey & Frenkel, 1998; Kijlstra et al., 2008). Felid species are the only hosts that are able to shed *T. gondii* oocysts in the environment (Dubey, 2016) (Nicolle & Manceaux, 1908). However, the parasite is present in a wide range of warm blooded animals, first as tachyzoites and later as bradyzoites, also called tissue cysts (Dubey, 2016). When a cat consumes an infected intermediate host, the parasite can complete its lifecycle (Dubey, Miller, & Frenkel, 1970). There are a couple publications on *T. gondii* in rodent and insectivore species in the Netherlands. In 2012 250 small mammals were tested and found 4% positive for *T. gondii *(Meerburg, Craeye, Dierick, & Kijlstra, 2012). Another study form the Netherlands found 11.9% of the rodents and insectivores (*n* = 101) positive for *T. gondii *(Kijlstra et al., 2008). Research from 2014 on common moles (insectivore) from the Netherlands found a prevalence of 2.3% (*n* = 86)(Krijger et al., 2014). It is interesting to see that the prevalence varies per species and even per location.

Because rodents can be host to both zoonotic pathogens *Leptospira* spp. (definitive host) and *T. gondii* (intermediate host), and since the current status of its prevalence in the Netherlands remains unknown, rodents and insectivores from several geographically spread areas in the Netherlands were tested on the presence of those two zoonotic pathogens.

## 2 METHODS

**Figure 1:**
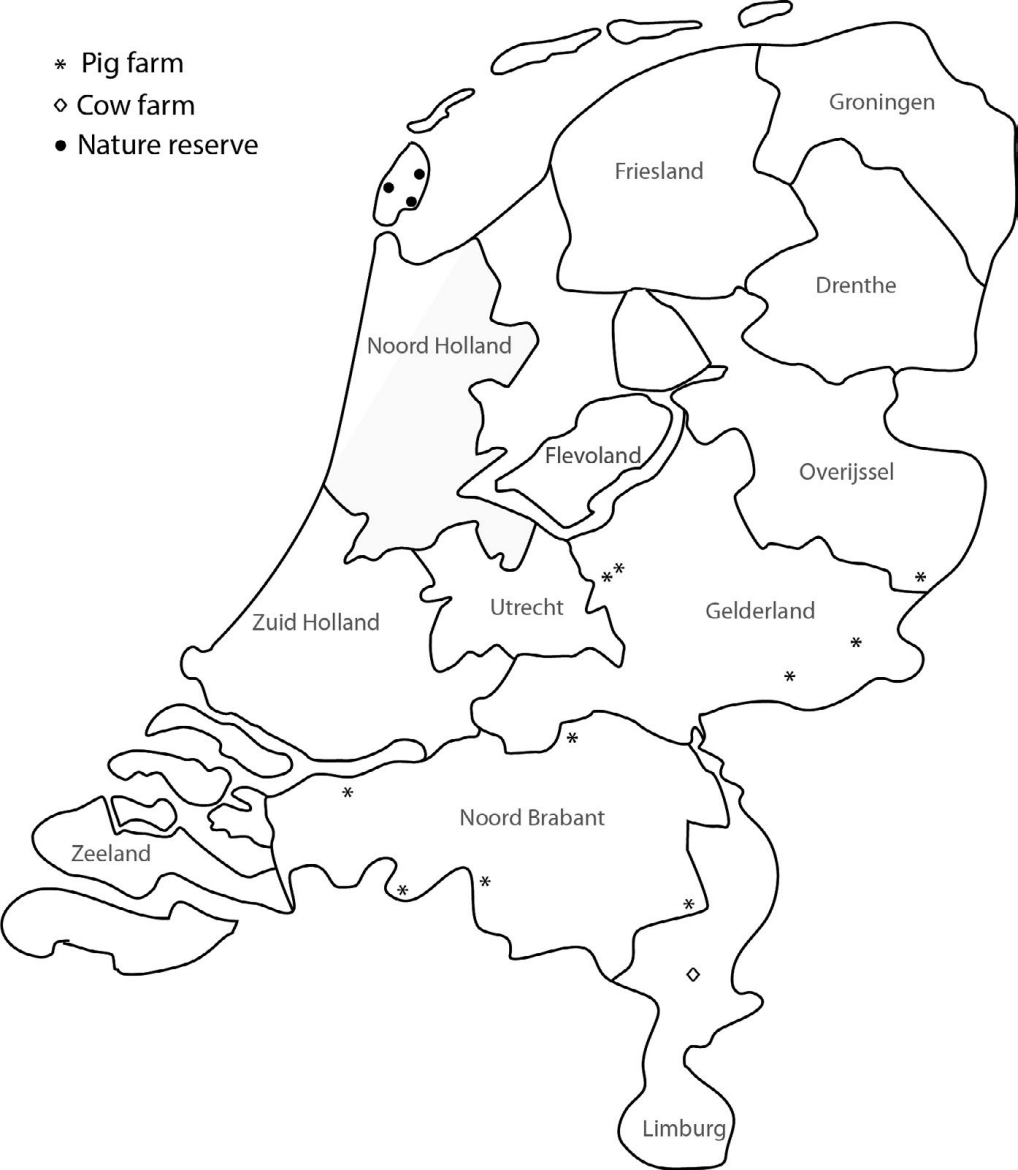
Map of The Netherlands showing rodent trapping locations. * is a pig farm, ◊ is a cow farm and ● a nature reserve

Rodent trapping was conducted from November 2016 until January 2017 on 10 conventional pig farms and one cow farm, distributed over four provinces in the Netherlands; Limburg, Noord‐Brabant, Gelderland and Overijssel (Figure 1) by professional and certified rodent management companies. Each farmer was surveyed and asked about the presence of cats and/or stray cats on their farm. All locations were visited and screened for rodent tracks. Snap‐traps were then placed accordingly by a certified pest‐manager. Traps were placed one week in pre‐bait position, after which they were placed and used for 1 month. Traps were checked upon daily to ensure a maximum period between capture and storage of 24 hr. Trapped animals were stored in separate seal bags at −18°C.

In October 2018, rodents were trapped on three locations on recreational areas in nature reserves on the island Texel (province of Noord Holland, Figure 1) by a rodent manager using the EKO1000 traps, and by use of the rodenator (Meyer Industries). Trapped animals were stored in separate seal bags at −18°C.

All rodents were thawed at 4°C 24 hr before dissection. During dissection at Wageningen Bioveterinary Research (WBVR) each animal was identified to species level and sexed and of each rodent randomly one kidney and the brains were collected. Samples were stored at −20°C until further analysis. All rodent samples were tested for *Leptospira* spp. (*n* = 379), whereas the samples from rodents trapped on pig farms and Texel were besides *Leptospira* spp. also tested on *T. gondii* (*n* = 312).

### 2.1 *Leptospira* spp. diagnostics

**Figure 2:**
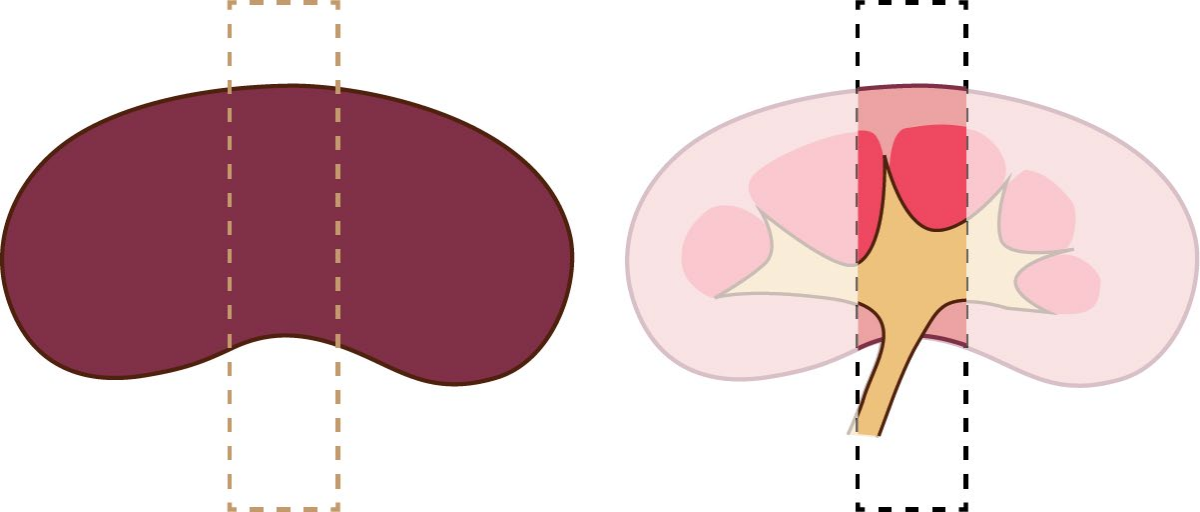
Schematic overview of the transversal slice of the kidney

From each kidney sample a small transversal slice (≤25 mg) was cut (Figure 2) and treated for DNA extraction (QIAamp DNA Mini Kit, QIAGEN). All tissues were processed for DNA extraction according to the manufacturer's protocol with some modifications; tissues were digested by using 360 μl of buffer ATL (QIAGEN) and 40 μl of proteinase K (QIAGEN), mixed and incubated for 3 hr at 56°C, were heated at 70°C for 10 min after adding AL buffer, after which ethanol was added. All DNA samples were stored at −20°C until further testing by PCR.

### 2.2 Quantitative polymerase chain reaction (RT‐qPCR) for *Leptospira* spp. detection and speciation

Each DNA sample was diluted (1:10) with UltraPure DNase/RNase‐free distilled water (Invitrogen) and tested in triplicate. The SYBR Green real‐time qPCR targetting *secY* gene was used (Ahmed, Engelberts, Boer, Ahmed, & Hartskeerl, 2009). Reactions of in total 25 μl were set up with 10 μl sample to be examined, 12.5 μl of SYBR Green Supermix (Bio‐Rad) of 2× stock reagent (100 mM KCl, 40 mM Tris‐HCI, pH 8.4, 0.4 mM of each dNTP, 50 units/ml iTaq DNA polymerase, 6 mM MgCl_2_, 20 nM fluoresein and stabilizers), 1 μl SecYIVF (400 nM) as forward primer and 1 μl SecYIV (400 nM) as reverse primer, 0.5 μl UltraPure DNase/RNase‐free distilled water (Invitrogen). For the negative control 10 μl sterile UltraPure water was used as template. A Bio‐Rad CFX96 real‐time PCR fast detection system was used to perform the reactions by a first cycle of 5 min of activation at 95°C with subsequent dissociation steps consisting of: 95°C/5 s; 54°C/5 s; 72°C/15 s for 40 cycles. The programme finished with 95°C/1 min and a cooling at 20°C/1 min and the dissociation was measured stepwise, every 0.5°C.

Amplicon specificity was checked by conducting a melting curve analysis which was also used to determine the *Leptospira* species; a sample was classified positive when Ct value ≤ 35 cycles and Tm between 78.5–84.5°C. Samples were tested in triplicate and classified as positive when ≥2 runs resulted positive. A retest in trifold was conducted on samples that gave only one amplification curve. Samples were classified as positive if the repeat run resulted in ≥1 positive reaction and if the amplification melting curve was conform to set values.

### 2.3 *Toxoplasma gondii* diagnostics

The brain tissue was thawed at 4°C. Samples were homogenized for 30 s by an ultra turrax homogenizer after adding 1 ml DPBS. DNA was extracted from 250 μl of the homogenized brain tissue with the DNeasy Blood & Tissue kit (Qiagen GMBH). The manufacturers’ protocol was slightly adjusted; 50–100 pc glass homogenizer beads were added to each sample and the samples were mixed by vortexing for 10 min at 220 *g* to facilitate lysis. Hereafter, lysis buffer was added and samples were then incubated for 2.5 hr at 56°C, after which another vortexing cycle of 10 min at 220 *g* took place. During the addition of ethanol, we added 1.5 µl HCl 35%, and used only 50 µl AE buffer to elute the DNA. DNA samples were stored at −20°C until tested by Real‐Time PCR. Of each sample 5 µl DNA was tested by a RT‐qPCR using SYBR Green (Applied Biosystems) in an ABI 7500 Real‐Time PCR system (PE Applied Biosystems). A standard reaction mixture contained 12.5 µl of SYBR Select Master Mix, 1 µl (10 µM) of the primers, 5 µl of DNA template and 5.5 µl PCR grade water. The primers (529‐F: AGG AGA GAT ATC AGG ACT GTA G and 529‐R: GCG TCG TCT CGT CTA GAT CG) are complementary to the 529‐bp repeat element (GenBank AF146527). The cycling profile involved an initial PCR activation step at 95°C for 10 min, followed by 40 cycles of denaturation at 95°C for 15 s and primer annealing and extension at 60°C for 60 s. Following amplification, a melt curve analysis was performed to verify the specificity of the amplified products by their specific melting temperatures (Tm). For quantification of the amount of *T. gondii* DNA in the samples, a standard curve of DNA extracted from cultured tachyzoites from the *T. gondii* RH strain was used. Data acquisition and analysis of the results were performed using the 7500 System SDS Software (Applied Biosystems). Samples with Ct‐value < 37.5 and Tm‐value between 81.9 and 83.5°C were considered as positive.

### 2.4 Statistical analysis

To compare frequency between sex the Chi‐square test was used, to analyse between provinces a one‐way ANOVA was used, for further analyses descriptive statistics were used. Results were considered statistically significant with a *p*‐value of *p* < .05. Statistical analyses were performed using SPSS, version 23 (IBM SPSS Statistics Inc).

## 3 RESULTS

In total 379 rodents and insectivores were trapped, 351 on livestock farms (Limburg, Brabant, Gelderland and Overijssel), and 28 in nature reserves (Noord‐Holland). The trapped animals consisted out of three insectivore and seven different rodent species. About half of the number of animals were black rats (*Rattus rattus*, 49.6%), second predominant species was the house mouse (*Mus musculus*, 22.2%). All trapped animals were tested for pathogenic *Leptospira* spp*.* Twenty were found positive (*Leptospira* species *Interrogans* (*n* = 15) and *Kirschneri* (*n* = 5)) thus showing an overall incidence of 5.3% (Table 1). The prevalence of *Leptospira* spp. among wild rodents and insectivores differs significantly per province (*p* = .006), with Gelderland being the province with the highest incidence (Table 2). There was no significant association between rodent sex and *Leptospira* spp. infection (*p* = .85).

**Table 1:** Infection percentage of rodent species with *Leptospira* and *Toxoplasma gondii*

Mammal species	Rodent or insectivore	No. positive/total (%)	
		*T. gondii*	*Leptospira*
Wood mouse (*Apodemus sylvaticus*)	Rodent	0/19 (0)	2/19 (10.5)^a^
Harvest mouse (*Micromys minutus*)	Rodent	0/1 (0)	0/1 (0)
Common vole (*Microtus arvalis*)	Rodent	0/8 (0)	2/8 (25.0)^b^
Common house mouse (*Mus musculus*)	Rodent	0/84 (0)	5/84 (6.0)^c^
Muskrat (*Ondatra zibethicus*)	Rodent	0/1 (0)	1/1 (100)^a^
Brown rat (*Rattus norvegicus*)	Rodent	5/36 (13.8)	5/66 (7.6)^a^
Black rat (*Rattus rattus*)	Rodent	0/151 (0)	1/188 (0.5)^b^
Greater white‐toothed shrew (*Crocidura russula*)	Insectivore	0/2 (0)	0/2 (0)
Common shrew (*Sorex araneus*)	Insectivore	0/9 (0)	4/9 (44.4)^c^
Crowned shrew (*Sorex coronatus*)	Insectivore	0/1 (0)	0/1 (0)
Total		5/312 (1.6%)	20/379 (5.3%)

a Species *Leptospira interrogans*.

b Species *Leptospira kirschneri*.

c Both species *Leptospira interrogans* (*Mus musculus* *n* = 4, *Sorex araneus* *n* = 3) and *kirschneri* (*Mus musculus* *n* = 1, *Sorex araneus* *n* = 1).

**Table 2:** *Leptospira* infection percentage of the tested small mammals per province

Province	No. tested animals	No. positive	Prevalence
Limburg	219	4	1.8%
Noord‐Brabant	66	7	10.6%
Overijssel	40	5	12.5%
Gelderland	26	4	15.4%
Noord Holland	28^a^	0	0%
Total	379	20	5.3%

a On Texel (Noord Holland), only brown rats were trapped (*n* = 28).

Five animals were found positive for *T. gondii* (1.6%, Table 1), of which three female and two male rats. All five were brown rats from Texel (Noord Holland). With 28 brown rats (17 females, 11 males) trapped on Texel, the prevalence of this group of rodents from this specific island comes to 17.9%.

## 4 DISCUSSION

Although research is conducted, still little is known about the presence and risks of zoonotic pathogens carried by rodents and/or insectivores zoonoses in the Netherlands. This knowledge gap limits opportunities for preventive measures and confounds the approximation of the potential transmission to humans. Until now, there is still little known and published about the presence of *Leptospira* spp. in rodents and insectivores in the Netherlands and other European countries. Therefore we tested rodents and insectivores from several geographically spread areas in the Netherlands on presence of those two zoonotic pathogens. In total, 5.3% of the animals (*n* = 379) tested positive for *Leptospira* DNA, and 1.6% of the animals (*n* = 312) tested were positive for *T. gondii* DNA. Our results show that insectivores and rodents might be used as an indicator for the environmental contamination and/or the contamination in wildlife for *Leptospira* spp.

Most studies focus on *Rattus norvegicus* only, because these animal carriers are recognized as important infection sources for humans (Aviat et al., 2009; Runge et al., 2013) and are often present near shores of lakes, canals and rivers. In this way, they pose a serious threat for surface water contamination. A study from France (Aviat et al., 2009) found 34.7% of the trapped brown rats (*n* = 36) positive for pathogenic *Leptospira* spp., and a study in Germany found 21% of the 586 brown rats positive (Runge et al., 2013). A recent study from the Netherlands reported an infection range of 33%–57% in brown rats (Maas et al., 2018). It is known that the infection rate among rats is highly variable in time and place (Kuiken, 1990; Kuiken, van Dijk, Terpstra, & Bokhout, 1991), which is also underpinned by the recent study in the Netherlands (Maas et al., 2018). We found a lower infection percentage in the small mammals tested (5.3%) than these European studies. This difference could be due to multiple factors, such as difference in diagnosis methods used, or trapping year, or season. Although the majority of publications use serological methods, it is important to use molecular detection, like in this study. A serious disadvantage of using serological methods for diagnosis is that it only detects the pathogens presence when there are sufficient levels of anti‐*Leptospira* spp. antibodies present (Ahmed, Grobusch, Klatser, & Hartskeerl, 2012; Musso & La Scola, 2013). Using serological assays could therefore might lead to incorrect results. However, the main reasons for the difference in infection percentages found is that studies mentioned above focus on *R. norvegicus* only, in contrast to our study which includes more animal species. Another important reason for the difference in infection percentages is the location where the mammals were trapped. The studies above all researched mammals trapped nearby water. The animals from our study are from farms and nature reserve areas and not on locations linked to water or water rich spaces such as rivers, canals or recreation lakes.

Although brown rats are considered the most important hosts spreading the bacterium to humans, almost every mammal might be reckoned as potential bearer and disseminator of *Leptospira* spp. (Hartskeerl, 2006; Mwachui et al., 2015). Therefore, this study was set up to test more animal species than brown rats only. In this study it is indicated that, even though with a lower abundance, pathogenic *Leptospira* spp. are also widely distributed in other small mammals; the prevalence of *Leptospira* spp. in the tested rodents and insectivores ranged between 1% and 15%, with an average of 5.3%. This is confirmed by literature from European countries which report on the occurrence of *Leptospira* spp. in small rodents and shrews. A study on *Leptospira* spp. in small rodents from Croatia tested 7% of the rodents positive (*n* = 227) (Turk et al., 2003). Research from Germany on small mammals found an incidence of 5.7% (*n* = 736) (Obiegala et al., 2017), which is in line with our findings. Another study from Germany (Obiegala et al., 2016) found an overall infection percentage of 9.7% (*n* = 2,961). A Swiss study from 2002 found leptospiral DNA in 12.6% of 190 small mammals (Adler, Vonstein, Deplazes, Stieger, & Frei, 2002). Czech research showed 11.6% of the trapped small mammals (*n* = 429) positive for pathogenic *Leptospira* spp., with infection ranges varying from 0% to 20% between species (Treml, Pejcoch, & Holesovska, 2002). We found both *L. interrogans* and *L*. *kirschneri* in the rodents population tested. It is remarkable that *L. kirschneri* was found in the *Rattus rattus* (black rat)*.* This black rat is worldwide associated with Icterohaemorrhagiae infections which belong to *L. interrogans* (Kuiken, 1990) although it harbours also *L. kirschneri* in Brazil and Mayotte (Desvars et al., 2012; Moreno et al., 2016). It can be concluded that besides seasonal, geographic and temporal factors, the host species also plays a role in the infection rate.

When looking at *T. gondii* in the trapped animals, all rodents and insectivores caught on the pig and cow farms tested negative for this parasite. This is not in line with the expectations since previous studies conducted on farms in the Netherlands found rodents as well as insectivore species carrying *T. gondii;* rodents and insectivores trapped on organic farms in the Netherlands in 2004 gave an infection rate with *T. gondii* of 4% (*n* = 250) among species; house mice (9.0%), common voles (4.2%) and white‐toothed shrews (2.0%) (Meerburg et al., 2012). Research from 2008 in the Netherlands on rodents from pig‐farms, found a prevalence of 11.9% (*n* = 101) in rodents and insectivores (Kijlstra et al., 2008). Prevalences differed among animal species, in descending order: 14.3% of *Apodemus sylvaticus* (*n* = 7) tested positive for *T. gondii,* 13.6% of the *Crocidura russula* (*n* = 22), 10.3% of the *Rattus norvegicu*s (*n* = 39) and 6.5% of the trapped *Mus musculus* (*n* = 31) (Kijlstra et al., 2008). As well in the study from Meerburg et al. (2012) as in the study by Kijlstra et al. (2008), it was noted that cats were present on the participating farms. Being the definitive host for *T. gondii,* cats could become infected by predation of infected intermediate hosts such as wildlife, or via ingestion of oocysts from the environment (Afonso, Thulliez, & Gilot‐Fromont, 2006; Afonso, Thulliez, Pontier, & Gilot‐Fromont, 2007; Hejlíček & Literak, 1998). In this study, however, all farms were free of cats, which might explain the absence of *T. gondii* in the small mammals tested. This is in contrast to the situation on the island Texel (NL) where there is a problem with stray cats (News, 2018; Spek, 2015). The presence of wild cats on this island (≈460 km^2^) could explain the relatively high prevalence of 17.9% among the trapped rodents (brown rats) from Texel.

Our study had some limitations, as the rodents and insectivores came from five provinces, while there are 12 provinces in the Netherlands. A suggestion for further research would be to collect (more) rodents and insectivores from over the whole country, including all provinces to get insight in high and low frequency areas. Another ‘limitation’ of the study is that the samples were tested using primers which could not detect mixed infections (Moseley et al., 2018), leading to a conclusion of the presence of maximal one *Leptospira* species per infected animal, whereas the animal could potentially be infected with multiple *Leptospira* species. For future research, the primers for testing mixed infections should be tested and if they work as described, they should be used.

In conclusion, the results of this study indicate that *Leptospira* spp. and *T. gondii* are present in the population of wild small mammals in the Netherlands, indicating the importance of the studies for these infectious agents. The presence of *Leptospira* spp. in rodents and insectivores living around farms, could lead to transmission of the bacterium to human food (livestock) of humans itself.

The presence of *T. gondii* in small rodents present around farms could be a risk factor as rodents tend to visit barns. Theoretically production animals such as pigs could then get acquire infection, leading to potential risk for human infection as the infected meat ends on our table, potentially raw or undercooked (Guo et al., 2015; Kijlstra & Jongert, 2009). Another very important risk factor for *T. gondii* is the presence of (stray) cats. A suggestion for further research would be to study the prevalence of *T. gondii* in (stray) cats in the Netherlands. For *Leptospira* spp. it is an interesting and important finding that not only brown rats, but both rodent and insectivore species are carriers, and therewith could be considered as potential sources for human leptospirosis in the Netherlands. Consequently, rodents and insectivores could be good indicator species for monitoring of the presence of these zoonotic pathogens in the environment.

## CONFLICT OF INTEREST

No conflict of interest exist.
